# Predicting mortality in patients diagnosed with advanced dementia presenting at an acute care hospital: the PROgnostic Model for Advanced DEmentia (PRO-MADE)

**DOI:** 10.1186/s12877-023-03945-8

**Published:** 2023-04-28

**Authors:** Palvinder Kaur, Palvannan Kannapiran, Sheryl Hui Xian Ng, Jermain Chu, Zhi Jun Low, Yew Yoong Ding, Woan Shin Tan, Allyn Hum

**Affiliations:** 1 Health Services and Outcomes Research, National Healthcare Group, 3 Fusionopolis Link, #03-08, Singapore, 138543 Singapore; 2 Department of Palliative Medicine, Tan Tock Seng Hospital, 11 Jalan Tan Tock Seng, Singapore, 308433 Singapore; 3 Geriatric Education and Research Institute, 2 Yishun Central 2, Singapore, 768024 Singapore; 4 Palliative Care Centre for Excellence in Research and Education, Tan Tock Seng Hospital, 10 Jalan Tan Tock Seng, Singapore, 308436 Singapore

**Keywords:** Advanced dementia, Acute care setting, Prognostication, One-year mortality, Palliative care

## Abstract

**Background:**

Challenges in prognosticating patients diagnosed with advanced dementia (AD) hinders timely referrals to palliative care. We aim to develop and validate a prognostic model to predict one-year all-cause mortality (ACM) in patients with AD presenting at an acute care hospital.

**Methods:**

This retrospective cohort study utilised administrative and clinical data from Tan Tock Seng Hospital (TTSH). Patients admitted to TTSH between 1st July 2016 and 31st October 2017 and identified to have AD were included. The primary outcome was ACM within one-year of AD diagnosis. Multivariable logistic regression was used. The PROgnostic Model for Advanced Dementia (PRO-MADE) was internally validated using a bootstrap resampling of 1000 replications and externally validated on a more recent cohort of AD patients. The model was evaluated for overall predictive accuracy (Nagelkerke’s R^2^ and Brier score), discriminative [area-under-the-curve (AUC)], and calibration [calibration slope and calibration-in-the-large (CITL)] properties.

**Results:**

A total of 1,077 patients with a mean age of 85 (SD: 7.7) years old were included, and 318 (29.5%) patients died within one-year of AD diagnosis. Predictors of one-year ACM were age > 85 years (OR:1.87; 95%CI:1.36 to 2.56), male gender (OR:1.62; 95%CI:1.18 to 2.22), presence of pneumonia (OR:1.75; 95%CI:1.25 to 2.45), pressure ulcers (OR:2.60; 95%CI:1.57 to 4.31), dysphagia (OR:1.53; 95%CI:1.11 to 2.11), Charlson Comorbidity Index ≥ 8 (OR:1.39; 95%CI:1.01 to 1.90), functional dependency in ≥ 4 activities of daily living (OR: 1.82; 95%CI:1.32 to 2.53), abnormal urea (OR:2.16; 95%CI:1.58 to 2.95) and abnormal albumin (OR:3.68; 95%CI:2.07 to 6.54) values. Internal validation results for optimism-adjusted Nagelkerke’s R^2^, Brier score, AUC, calibration slope and CITL were 0.25 (95%CI:0.25 to 0.26), 0.17 (95%CI:0.17 to 0.17), 0.76 (95%CI:0.76 to 0.76), 0.95 (95% CI:0.95 to 0.96) and 0 (95%CI:-0.0001 to 0.001) respectively. When externally validated, the model demonstrated an AUC of 0.70 (95%CI:0.69 to 0.71), calibration slope of 0.64 (95%CI:0.63 to 0.66) and CITL of -0.27 (95%CI:-0.28 to -0.26).

**Conclusion:**

The PRO-MADE attained good discrimination and calibration properties. Used synergistically with a clinician’s judgement, this model can identify AD patients who are at high-risk of one-year ACM to facilitate timely referrals to palliative care.

**Supplementary Information:**

The online version contains supplementary material available at 10.1186/s12877-023-03945-8.

## Background

Dementia is a progressive disease that places significant burden on individuals, families, and society. In 2019, dementia was estimated to affect 57 million individuals globally and is projected to triple by 2050 [1]. With population ageing, the overall burden of advanced dementia (AD) will increase worldwide. Globally, the place of care and death for patients with dementia varies widely [2, 3]. In many higher-income countries such as Belgium, England, Netherlands, and the United States of America [2], long-term-care (LTC) facilities such as nursing homes or care homes are common places of care and death for persons with dementia. In countries where the LTC sectors are less developed, the acute care setting is the default route in the pursuit of dementia care even at advanced stages of the illness [2]. In Singapore, 69% of AD patients died in the hospital [4].

Dementia is often not perceived to be a terminal illness. With an estimated median survival of 1.3 years [5], the end-of-life experience of individuals dying from AD is often characterised by severe functional impairment, high symptom burden, frequent hospitalizations and substantial healthcare costs which adds to caregiver distress [4–7]. Despite having a similar symptom burden to patients with advanced cancer [8], many patients with dementia do not receive palliative care in a timely manner [9–11]. In Singapore, one in two patients with AD were referred to palliative home care services in the last one-month of life. [11].

People with dementia and their caregivers would benefit from palliative care due to the terminal nature of dementia and complex care requirements [11, 12]. To enable timely access to palliative care, the European Association of Palliative Care has highlighted the importance of prognostication and timely recognition of dying [13]. While referrals to palliative care should be made based on patients’ needs and goals of care [14], accurate prognostication can help to triage AD patients who are at high-risk of short-term mortality in countries or settings where there are limited resources. Due to the protracted and unpredictable decline in the disease trajectory, recognising the terminal phase is a challenge for patients with AD [3, 15, 16].

Standardised mortality risk estimates could help with resource planning for end-of-life care. Current tools for estimating prognosis are limited in terms of reliability in producing accurate prognostic estimates. While there are several models to estimate prognosis in patients with dementia [17], only three models have been specifically developed for AD [18–20]. All three models sought to predict six-months to one-year mortality among AD patients residing in a LTC facilities or receiving home hospice care. Due to the specific populations of interest, these models may not be generalisable to patient populations receiving care in other healthcare settings such as the acute care hospitals [18–20]. With the exception of Hsieh et al. [19], the models demonstrated poor predictive performance [20, 21]. External validation of these prognostic models is scarce.

Despite the substantial number of dementia patients receiving care in the acute care setting, there is a dearth of prognostic models developed to predict mortality among AD patients in the acute care setting. It is imperative that predictive algorithms are developed and validated comprehensively in order to establish robustness of the model prior to use in the clinical setting. With evidence-informed selection of variables based on our review of the literature [22], guided by clinical judgement and the application of best practices recommended for model development and evaluation [23, 24], we aim to develop and validate a prognostic model to predict one-year mortality among AD patients presenting at the acute care setting.

## Methods

### Study setting

Singapore’s public healthcare system is organised into three integrated regional healthcare systems (RHS) – the National Healthcare Group (NHG), Singapore Health Services (SHS) and National University Health Systems (NUHS). Nearly 80% of all acute care services and 20% of primary care are provided by the three RHSs.

The NHG provides healthcare services to an estimated population size of 1.5 million residents through an integrated network of institutions that includes two public restructured hospitals, six primary care polyclinics and two national healthcare centres. This study sample comprised of patients who were admitted to Tan Tock Seng Hospital (TTSH), the largest publicly funded acute general hospital for the NHG cluster and the second largest hospital in Singapore with over 1,500 beds.

### Study design and population

We adopted a retrospective cohort study design to predict one-year mortality among patients diagnosed with AD presenting at an acute care hospital. We employed a two-step strategy to identify patients with advanced dementia. First, patients with dementia were identified based on the primary and secondary diagnoses of dementia using the International Classification of Diseases, tenth revision, clinical modification (ICD-10-CM) codes (listed in **Additional File 1 Table**S1) among patients admitted to TTSH between 1st July 2016 and 31st October 2017. Second, dementia patients were denoted as having AD if they presented with characteristics consistent with the Functional Assessment Staging Tool (FAST) Stage 7 [25], had severe cognitive impairment defined as a mini-mental state examination (MMSE) score ≤ 10 or had a clinical diagnosis of AD in the medical notes. As the FAST tool was not commonly used then in our clinical setting, the FAST staging was retrospectively applied based on information recorded in the clinical notes (**Additional File 1 Table**S2). This cohort of AD patients formed the analytical sample used in the development of the model. Details of this two-step identification strategy is available in **Additional File**1.

Patients who were referred to palliative care, inpatient or home hospice, and had no clinical diagnosis of advanced dementia were excluded from the analysis.

### Data sources

Patients’ demographics, clinical conditions, laboratory tests, treatments, and referrals to other healthcare services at the time of AD diagnosis were retrieved from electronic health records (Computerised Patient Support System 2.0) at TTSH. Healthcare services utilised at both acute [i.e., inpatient admissions (IP), emergency department (ED)] and non-acute [specialist outpatient clinics (SOC), and polyclinics] settings within the NHG health system, Charlson Comorbidity Index (CCI) and death dates were extracted from the RHS database. Briefly, the RHS database is a research database that consists of administrative and diagnostic data across institutions within the NHG health system. Details of this database have been previously published [26].

### Potential prognostic variables

Adopting an evidence-informed approach, the selection of potential prognostic variables was guided by clinical inputs and the results of a scoping review undertaken by the study team previously [22]. We followed the methodological steps for conducting scoping reviews as outlined by Arksey and O’Malley [27] with advancements made by Levac et al. [28]. A total of 239 variables influencing mortality in dementia patients were identified and categorised broadly into six domains: individual factors, functional ability, health status, cognition and mental health, disease modifying treatments and health system factors. Definitions of these six domains can be found in Kaur et al. [22].

We mapped the variables that were identified from our review to the data that was available in TTSH’s electronic medical health records and the RHS database. A total of 37 potential prognostic variables across the six domains were identified (Table 1).

**Table 1:** Potential prognostic variables to predict one-year mortality among AD patients

Domain	Variables
**(1) Individual factors**	Age, gender, marital status, ethnicity, housing type, living situation, presence of caregiver, documentation of advance care plans (ACP)
**(2) Health status**	Type of dementia, FAST stage, Charlson’s Comorbidity Index (CCI), pneumonia, pressure ulcers, biochemical tests (sodium (mmol/L), potassium (mmol/L), haemoglobin (mmol/L), white blood cells (X 10^9^/L), urea (mmol/L) and serum albumin (g/dL))
**(3) Function**	Mobility impairment requiring use of aid, presence of dysphagia, functional dependency of four or more activities of daily living (ADL)
**(4) Cognitive and mental health**	History of depression, anxiety, mood disorders or other conditions related to mental health and history of delirium, history of behavioural and psychological symptoms of dementia (BPSD) and history of agitation
**(5) Treatment**	Use of enteral tube, prescribed with psychotropic medication, opioids, memantine or acetylcholinesterase inhibitors (ACEi)
**(6) Health system factors**	Referrals to community or home-based programs, IP, ED, SOC, Polyclinic, and average length-of-stay (ALOS)

The baseline of this study was defined as date of diagnosis of AD, which was the date at which symptoms of AD were first identified or recorded in either the inpatient or outpatient medical notes. Potential prognostic variables available at study baseline were extracted. Variables measuring impairment in Activities of Daily Living (ADL) such as feeding, dressing, bathing, toileting, transferring and ambulating were extracted. Biochemical tests included blood investigations performed to obtain readings for sodium (mmol/L), potassium (mmol/L), haemoglobin (mmol/L), white blood cells (X 10^9^/L), urea (mmol/L) and serum albumin (g/dL) levels. Baseline biochemical data were defined as readings dated within three days of the inpatient admission or outpatient episode during which the AD diagnosis was made. Biochemical readings were categorised as normal and abnormal levels according to clinically relevant thresholds. Healthcare utilisation was defined as all utilisation that occurred one year prior to AD diagnosis. Details on specifications of variables and time of extraction can be found in **Additional File**2.

### Outcome

The primary outcome of interest was defined as all-cause mortality within 365 days of AD diagnosis. Decedents were defined as those who died within one-year of AD diagnosis and survivors were defined as those who were alive within one-year of AD diagnosis.

### Sample size calculation

We computed the minimum sample size based on a rule of thumb stating that a minimum of 10 events per variable was required for the development of the model [29]. Assuming (i) 20 predictors per model, (ii) a minimum of 10 events per variable over a one-year period and (iii) hospital mortality rate of 20%, we needed a minimum sample size of 1000 patients diagnosed with AD for this study.

### Statistical methodology

We followed the recommendations and guidelines for development and validation of clinical prediction models by Harrell [30] and Steyerberg [23] in our analytical approach. For the reporting of the model diagnostics and results, the recommendations and guidelines provided by the Transparent Reporting of a Multivariable Prediction Model for Individual Prognosis or Diagnosis (TRIPOD) statement were adhered to [24].

#### Descriptive analysis

We compared baseline characteristics between decedents and survivors. Continuous and categorical data were analysed using t-tests and Chi-square tests and reported as mean ± standard deviation (SD) and column percentages respectively. A *p* value of < 0.05 was considered to be statistically significant. Within each domain, potential prognostic variables were analysed for multi-collinearity. No collinearity was observed across all variables (data not shown).

#### Missing data

To better understand the mechanism of missingness in the data, patients with complete and missing data were compared using univariate analysis and logistic regression [31]. Data was assumed to be missing at random because patients with missing data, specifically for biochemical readings, were more likely to have been diagnosed with AD in the outpatient setting. Biochemical tests had the highest proportion of missing values (range: 10.7–20.6%) (**Additional file 3 Table**S1). Multiple imputation using predictive mean matching was adopted as it quantifies the uncertainty of missing values by generating multiple different plausible datasets based on the observed values [30]. A total of 50 datasets were generated and each dataset was analysed separately. Model coefficients were pooled and averaged using Rubin’s rules [31]. Performance measures were averaged across the 50 datasets. Further details on missing data and imputation can be found in **Additional File**3.

#### Model development and internal validation

There were three key steps in the construction of this predictive algorithm – model development, internal validation, and external validation.

Model development is the process that leads to the final prediction equation. The associations between the potential prognostic variables and mortality were assessed using multivariable logistic regression. Time-to-event methods such as Cox proportional hazards modelling was not used as outcome data was available for all patients and no patients were censored at the end of the one-year mortality risk period. Backward variable selection was undertaken to retain variables associated with mortality based on statistical significance (p < 0.05) [23, 32].

Internal validation aims to determine if model performance is reproducible in the same underlying population used for model development. As prognostic models can be expected to perform better in datasets used for its development, we aimed to estimate the extent of optimism, or the difference in model performance on the original dataset and on resampled datasets. Bootstrap resampling of 1000 replications for each imputed dataset was performed. [23, 32]. For each performance measure, the average estimated optimism across all imputed datasets was recorded. The optimism was then subtracted from the performance measures to derive optimism-adjusted estimates.

#### External validation

External validation evaluates the generalisability of the final model in a new dataset that was not used in its development. A temporal validation approach was undertaken where a more recent cohort of AD patients were identified from the same hospital [33]. Patients admitted to TTSH between 1st January 2018 and 31st December 2018 were identified. The two-step strategy as described in **Additional File**1 was used to identify patients with AD for this analysis. Accounting for the nine variables that were included in the final model, a minimum of 10 events per variable over a one-year period and a hospital mortality rate of 20%, a sample size of 450 was required to power the analysis. Missing data was imputed using predictive mean matching and ten datasets were generated. The final model derived from the development cohort was applied to the external cohort’s imputed datasets. The model’s performance measures were averaged across the ten datasets and were reported accordingly.

#### Model performance measures

Model performance was evaluated based on overall measures of predictive accuracy, discrimination, and calibration [23].

Overall predictive accuracy was assessed using Nagelkerke’s R^2^ and Brier’s score. The Nagelkerke’s R^2^ measures the explained variation of the model, and ranges from 0 to 1, with higher values indicating a better fit of the model. The Brier’s score, which ranges from 0 to 1, measures the mean squared prediction error between predicted probabilities and observed values [23]. Smaller values of the Brier’s score (closer to zero) denote more accurate prediction [23].

Discrimination refers to the ability to differentiate between those who will die within one-year of AD diagnosis and those who will not. Discriminative power of the model was determined by measuring the area-under-the-curve (AUC). An AUC of 0.5 would mean predictions were no better than random and a value of 1 would represent perfect discrimination between patients with and without the outcome. In general, an AUC of 0.70 to 0.80 is indicative of good discrimination [34].

Calibration is the agreement between predicted risk probabilities produced by the model and observed mortality risk [23, 35]. The calibration slope was determined by regressing the observed mortality risks on predicted mortality risk probabilities. A slope of < 1 suggests that the model predictions were overestimated for patients who are at high risk and underestimated for those who are at low risk, and a slope of > 1 would indicate the opposite [36]. Calibration-in-the-large (CITL) is a basic measure to determine mean calibration and is estimated from the intercept of the regression curve. Negative values suggest an overestimation of predicted risks while positive values suggest an underestimation of the predicted risks. A slope of 1 and a CITL of 0 is considered ideal [23].

In addition, we examined the practical application of the final model to predict one-year mortality in this population by computing the sensitivity, specificity, positive predictive value (PPV) and negative predictive value (NPV) based on varying probability thresholds. Sensitivity was defined as the proportion of patients who died within one-year of AD diagnosis and was correctly identified as high risk. Specificity was defined as the proportion of patients who did not die within one-year of AD diagnosis and was correctly classified as low risk. The PPV was defined as the proportion of patients who were classified as high risk and died within one-year of AD diagnosis. The NPV was defined as the proportion of patients who were classified as low risk and did not die within one-year of AD diagnosis.

All models were developed in STATA version 17. STATA packages ‘fitstat’ was used to determine Nagelkerke’s R^2^ and ‘pmcalplot’ function was used to obtain calibration plots.

### Ethics approval

All data were de-identified and anonymised by an independent third party. Research ethics approval was obtained from the National Healthcare Group Domain Specific Review Board (NHG DSRB: 2018/00876).

## Results

### Baseline characteristics

A total of 1,077 patients were included in the developmental cohort. Within one-year of AD diagnosis, 318 (29.5%) patients died. Overall, the mean age of this population was 85 years (SD: 7.7 years), with 64.2% being female, and 87.2% being ethnically Chinese. In the developmental cohort, 87.4% and 12.6% were diagnosed with AD in the inpatient and outpatient setting respectively.

Table 2 describes the differences in baseline characteristics between survivors and decedents within one-year of AD diagnosis by each domain. In terms of individual factors, compared to survivors, decedents were significantly older, were more likely to be of the male gender and had more non-familial caregiver support.

**Table 2:** Development cohort - comparison of survivors and decedents within one-year of AD diagnosis

Characteristics	Survivors (n = 759)	Decedents (n = 318)	*P* value
**(1) Individual Factors**			
**Age > 85 years (n, col %)**	344 (45.3)	211 (66.3)	**< 0.001**
**Female (n, col %)**	502 (66.1)	190 (59.7)	**0.04**
**Marital Status (n, col %)**			
Single	28 (3.7)	8 (2.5)	
Married	548 (72.2)	228 (71.7)	0.70
Widowed/divorced	146 (19.2)	68 (21.4)	
Missing data	37 (4.9)	14 (4.4)	
**Ethnicity (n, col %)**			
Chinese	656 (86.4)	283 (89.0)	
Malay	42 (5.5)	9 (2.8)	0.27
Indian	54 (7.1)	22 (6.9)	
Others	7 (0.9)	4 (1.3)	
**Housing type (n, col %)**			
Nursing home	156 (20.6)	72 (22.6)	
Rental/1 to 2 room public flat	12 (1.6)	11 (3.5)	0.16
3 to 5 room public flat	472 (62.2)	199 (62.6)	
Private property	119 (15.7)	36 (11.3)	
**Living situation (n, col %)**			
Alone	39 (5.1)	12 (3.8)	
Family or friends	546 (71.9)	231 (72.6)	0.46
Nursing home	156 (20.6)	71 (22.3)	
Missing data	18 (2.4)	4 (1.3)	
**Presence of caregiver (n, col %)**			
Familial	156 (20.5)	54 (17.0)	
Non-familial	513 (67.6)	242 (76.1)	**0.01**
Both	19 (2.5)	8 (2.5)	
Missing data	71 (9.4)	14 (4.4)	
**Documentation of ACP (n, col %)**	257 (33.9)	120 (37.7)	0.22
**(2) Health Status**			
**Dementia type (n, col %)**			
Alzheimer’s disease	235 (31.0)	100 (31.5)	
Vascular disease	184 (24.2)	89 (28.0)	
Mixed dementia disease	175 (23.1)	72 (22.6)	0.38
Dementia disease	151 (19.9)	49 (15.4)	
Others	14 (1.8)	8 (2.5)	
**FAST stage (n, col %)**			
7a/b	26 (3.4)	6 (1.9)	
7c	692 (91.2)	304 (95.6)	0.07
7 d/e/f	8 (1.1)	3 (0.9)	
Undetermined	33 (4.3)	5 (1.6)	
**CCI (n, col %)**			
<8	340 (44.8)	100 (31.4)	
≥8	390 (51.4)	211 (66.3)	**< 0.001**
Missing data	29 (3.8)	7 (2.2)	
**Pneumonia (n, col %)**			
Yes	128 (16.9)	121 (38.1)	**< 0.001**
No	631 (83.1)	197 (61.9)	
**Pressure ulcers (n, col %)**			
Yes	32 (4.2)	59 (18.6)	
No	714 (94.1)	255 (80.2)	**< 0.001**
Missing data	13 (1.7)	4 (1.3)	
**Haemoglobin (mmol/L) (n, col %)**			
Normal (13.6 to 16.6)	96 (12.6)	28 (8.8)	
Abnormal (< 13.6; >16.6)	570 (75.1)	264 (83.0)	**0.02**
Missing data	93 (12.3)	26 (8.2)	
**White blood cells (X10** ^**9**^ **/L) (n, col %)**			
Normal (4 to 10)	370 (48.7)	151 (47.5)	
Abnormal (< 4; >10)	296 (39.0)	141 (44.3)	0.08
Missing data	93 (12.3)	26 (8.2)	
**Sodium (mmol/L) (n, col %)**			
Normal (135 to 145)	431 (56.8)	181 (56.9)	
Abnormal (< 135; >145)	241 (31.7)	109 (34.3)	0.38
Missing data	87 (11.5)	28 (8.8)	
**Potassium (mmol/L) (n, col %)**			
Normal (3.0 to 4.5)	550 (72.5)	216 (67.9)	
Abnormal (< 3.0; >4.5)	119 (15.7)	74 (23.3)	**0.01**
Missing data	90 (11.9)	28 (8.8)	
**Urea (mmol/L) (n, col %)**			
Normal (2.5 to 7.5)	398 (52.4)	108 (34.0)	
Abnormal (< 2.5; >7.5)	257 (33.9)	181 (56.9)	**< 0.001**
Missing data	104 (13.7)	29 (9.1)	
**Albumin (g/dL) (n, col %)**			
Normal (≥ 23g/dL)	563 (74.2)	221 (69.5)	
Abnormal (< 23g/dL)	23 (3.0)	48 (15.1)	**< 0.001**
Missing data	173 (22.8)	49 (15.4)	
**(3) Function**			
**Mobility impairment requiring use of aid (n, col %)**			
Yes	469 (61.8)	140 (44.0)	**< 0.001**
No	69 (9.1)	9 (2.8)	
Bedbound	221 (29.1)	169 (53.1)	
**Presence of dysphagia (n, col %)**	320 (42.2)	204 (64.2)	**< 0.001**
**Number of dependent ADLs (n, col %)**			
< 4	560 (73.8)	171 (53.8)	
≥ 4	172 (22.7)	143 (45.0)	**< 0.001**
Missing data	27 (3.5)	4 (1.2)	
**(4) Cognitive and mental health**			
**History of depression, anxiety, mood disorders and other conditions related to mental health (n, col %)**	85 (11.2)	30 (9.4)	0.39
**History of delirium (n, col %)**	188 (24.8)	113 (35.5)	**< 0.001**
**History of BPSD (n, col %)**	197 (26.0)	79 (24.8)	0.70
**History of agitation (n, col %)**	93 (12.3)	33 (10.4)	0.38
**(5) Treatment**			
**Use of enteral tube (n, col %)**			
Yes	95 (12.5)	69 (21.7)	
No	657 (86.6)	245 (77.0)	**< 0.001**
Missing data	7 (0.9)	4 (1.3)	
**Prescribed with psychotropic (n, col %)**	375 (49.4)	112 (35.2)	**< 0.001**
**Prescribed with opioids (n, col %)**	41 (5.4)	20 (6.3)	0.57
**Prescribed with ACEi (n, col %)**	46 (6.1)	6 (1.9)	**< 0.01**
**Prescribed with memantine (n, col %)**	54 (7.1)	11 (3.5)	**0.02**
**(6) Health system factors**			
**Referrals to community or home-based programs (n, col %)**	135 (17.8)	84 (26.4)	**0.001**
**Had IP (n, col %)**	653 (86.0)	298 (93.7)	**< 0.001**
**Had ED (n, col %)**	717 (94.5)	308 (96.9)	0.10
**Had SOC (n, col %)**	549 (72.3)	216 (67.9)	0.15
**Had Polyclinic (n, col %)**	328 (43.2)	122 (38.4)	0.14
**ALOS (days) (mean, SD)**	28.9 (39.3)	32.8 (33.3)	0.14

The bolded values are to indicate variables that had a statistically significant value defined as p<0.05.

Health status differed between both groups. When compared with survivors, the decedent group had a significantly higher proportion of patients with greater comorbidity burden, infections and abnormal biochemical values for haemoglobin, potassium, urea, and albumin.

Decedents had poorer overall function in contrast to survivors. More than half of decedents were bedbound and had dysphagia. Functional dependency for four or more ADLs were significantly higher in the decedent group versus the survivors.

The proportion of patients reporting cognitive and mental health issues were similar in both groups, with the exception of a history of delirium being reportedly higher in the decedent group.

Treatment variables varied between survivors and decedents. Compared to survivors, a significantly higher proportion of decedents were on enteral tubes. However, a lower proportion of decedents were prescribed psychotropic medications, ACEi and memantine.

The utilisation of healthcare services in the one-year prior to AD diagnosis was compared between the two groups. There were no differences in the proportion of patients who had ED, SOC, or polyclinic visits. While a higher proportion of decedents experienced at least one inpatient admission in the year prior, there was no difference in ALOS between the two groups. Upon AD diagnosis, more than a quarter of patients in the decedent group were referred to community or home-based programs compared to survivors.

### Final model

The multivariable logistic regression model demonstrated that age > 85 years, being male, having a pneumonia diagnosis, pressure ulcers, CCI ≥ 8, functional dependency for four or more ADLs, presence of dysphagia, as well as abnormal urea and albumin at the time of AD diagnosis were predictive of one-year mortality (Table 3).

**Table 3:** Predictors of one-year mortality among AD patients presenting at the acute care hospital

Predictors	Adjusted Odds Ratio	P	95% Confidence Interval
**Age (> 85 years)**	1.87	< 0.001	1.36 to 2.56
**Male**	1.62	< 0.001	1.18 to 2.22
**Pneumonia**	1.75	< 0.001	1.25 to 2.45
**Pressure ulcers**	2.60	< 0.001	1.57 to 4.31
**CCI ≥ 8**	1.39	0.04	1.01 to 1.90
**ADLs (≥ 4 dependent ADLs)**	1.82	< 0.001	1.32 to 2.53
**Presence of dysphagia**	1.53	0.01	1.11 to 2.11
**Abnormal urea (< 2.5 or > 7.5 mmol/L)**	2.16	< 0.001	1.58 to 2.95
**Abnormal albumin (< 23 g/DL)**	3.68	< 0.001	2.07 to 6.54
**Cons**	0.06	< 0.001	0.04 to 0.09

Final equation is as follows:

logit(p) = -2.77 + 0.62*Age>85years + 0.48*Male + 0.56*Pneumonia + 0.95*Pressure Ulcers + 0.33*CCI≥8 + 0.60*ADL≥4 functional dependencies + 0.43*Dysphagia+ 0.77*Abnormal Urea + 1.30*Abnormal Albumin

### Internal validation

For overall model performance, optimism-adjusted Nagelkerke’s R^2^ and Brier’s score were 0.25 (95% CI: 0.25 to 0.26) and 0.17 (95% CI: 0.17 to 0.17) respectively. The model demonstrated good discrimination, with optimism-adjusted AUC at 0.76 (95% CI: 0.76 to 0.76). Optimism-adjusted calibration slope and CITL was 0.95 (95% CI: 0.95 to 0.96) and 0 (95% CI: -0.0001 to 0.001) respectively. Calibration plots can be found in **Additional File**4.

Probability thresholds are used to classify patients as high-risk of death within one-year of AD diagnosis. Table 4 details the sensitivity, specificity, PPV and NPV for possible probability thresholds that could be operationalised in the clinical setting. If the model uses a probability threshold of 0.5, patients who have a mortality risk probability of ≥ 0.5 would be classified as high-risk of death within one-year of AD diagnosis. Based on this threshold, the model has a sensitivity of 36.87%, specificity of 92.62%, positive predictive value of 67.69%, and a negative predictive value of 77.79%.

**Table 4:** Sensitivity, specificity, PPV and NPV at varying probability thresholds*

Pr	Sensitivity (%)	Specificity (%)	PPV (%)	NPV (%)
0.9	2.14 (95% CI: 1.99 to 2.28)	99.92 (95% CI: 99.90 to 99.94)	93.21 (95% CI: 91.53 to 94.90)	70.90 (95% CI: 70.88 to 70.93)
0.8	7.96 (95% CI: 7.75 to 8.16)	99.40 (95% CI: 99.38 to 99.43)	84.82 (95% CI: 84.26 to 85.39)	72.05 (95% CI: 72.00 to 72.09)
0.7	15.65 (95% CI: 15.50 to 15.81)	98.13 (95% CI: 98.09 to 98.16)	77.79 (95% CI: 77.49 to 78.09)	73.52 (95% CI: 73.49 to 73.56)
0.6	28.01 (95% CI: 27.69 to 28.32)	95.42 (95% CI: 95.34 to 95.49)	71.92 (95% CI: 71.67 to 72.18)	75.98 (95% CI: 75.91 to 76.05)
0.5	36.87 (95% CI: 36.49 to 37.26)	92.62 (95% CI: 92.48 to 92.75)	67.69 (95% CI: 67.45 to 67.92)	77.79 (95% CI: 77.70 to 77.87)
0.4	51.18 (95% CI: 50.99 to 51.36)	85.59 (95% CI: 85.46 to 85.71)	59.81 (95% CI: 59.62 to 59.99)	80.71 (95% CI: 80.66 to 80.76)
0.3	64.64 (95% CI: 64.36 to 64.93)	73.93 (95% CI: 73.74 to 74.12)	50.96 (95% CI: 50.80 to 51.11)	83.31 (95% CI: 83.21 to 83.41)
0.2	81.41 (95% CI: 81.19 to 81.63)	51.46 (95% CI: 51.07 to 51.85)	41.28 (95% CI: 41.11 to 41.45)	86.86 (95% CI: 86.74 to 86.98)
0.1	98.07 (95% CI: 97.98 to 98.17)	17.20 (95% CI: 16.90 to 17.50)	33.17 (95% CI: 33.10 to 33.24)	95.53 (95% CI: 95.35 to 95.70)

*Proportion of AD patients who died within one-year: 29.5%

Pr: probability threshold; 95% CI: 95% confidence interval

### External validation

Of the 550 patients included in the external validation dataset, 145 (26.4%) patients died within one-year of AD diagnosis. The comparison of baseline characteristics between patients included in the development and external validation datasets can be found in **Additional File**5. Briefly, there was a higher proportion of patients who were diagnosed in the outpatient setting in the external validation dataset as compared to patients included in the development dataset (external cohort: 31.6% vs. developmental cohort: 12.6%, p value < 0.001). In comparison with patients in the model development cohort, patients in the external validation cohort had lower comorbidity, more pressure ulcers and better function (**Additional File**5).

We applied the final model to the external validation dataset. A decrease in model performance was observed. In terms of overall model performance, Brier’s score yielded an estimate of 0.18 (95% CI: 0.18 to 0.18). When externally validated, the model demonstrated an AUC of 0.70 (95% CI: 0.69 to 0.71), calibration slope of 0.64 (95% CI: 0.63 to 0.66) and CITL of -0.27 (95% CI: -0.28 to -0.26).

## Discussion

### Main findings

In this study, we developed and validated a prognostic model to predict one-year mortality among patients with AD receiving care in an acute care hospital. To the best of our knowledge, this is the first model in the literature that examined prognostication among AD patients presenting at an acute hospital. Our study found that being of age > 85 years, of the male sex, having pneumonia, pressure ulcers, CCI ≥ 8, functional dependency for four or more ADLs, dysphagia, abnormal urea, and abnormal albumin were predictive of one-year mortality among patients with AD in the acute care setting. The PRO-MADE demonstrated good discrimination and calibration properties at internal validation.

We saw a reduction in model performance when the final model was applied to a more recent cohort of AD patients in the external validation. As population profiles, diagnoses, treatments, and clinical practices change over time, predictions based on static data can become outdated, and hence no longer accurate [37, 38]. For a clinical prediction model to remain valid, it must evolve over time with continuous updates performed in a dynamic fashion [37]. Rather than build a new model, the recommended strategy would be to update, adjust or recalibrate the predictive model using the validation dataset [37, 38]. The updated model would combine the information captured during the model development phase with information from the new population [38]. The dissimilarity between the developmental and external cohort seen in this study could be attributed to differences in patient characteristics (**Additional File**5), changes in practice patterns or documentation of medical notes over time, warranting a recalibration of the existing model. After refitting the model and updating the coefficients of the prognosticators against one-year mortality in the external validation cohort, we managed to improve the model’s discriminative and calibration performance as seen in **Additional File**6.

### Comparison with other published prognostic models

We identified three published models which investigated the predictors of six to twelve months mortality of individuals living with dementia [18–20]. All three models focused on populations residing in LTC facilities and nursing homes as well as individuals who were receiving home hospice care services. Discriminatory performances based on AUC ranged from 0.65 to 0.81 [19–21], whereas the AUC of our model was 0.76. Different target populations and care settings rendered direct comparisons of model performances challenging. The higher discriminatory performance observed in the model by Hsieh et al. could be due to the focus on the prognostication of a group of AD patients at a more advanced stage of the disease (i.e., FAST Stage 7E and 7F) [19]. This may have resulted in less heterogeneity when estimating probability of death.

Predictors of mortality that were common across the three published models and our study include older age, male sex, greater comorbidity burden, and functional impairment as well as a diagnosis of pneumonia and presence of pressure ulcers. While dementia etiology was found to significantly predict mortality [20], we did not find this to be a differentiating factor in our study. At the advanced stages of the disease, mortality risks across the different dementia aetiologies may be attenuated. Furthermore, accuracy in the ascertainment and the documentation of dementia aetiology is not high in routine clinical practice. Misclassification may bias the magnitude of any associations towards the null.

Patients with AD may suffer from dyspnoea, eating problems, malnutrition, weight loss and incontinence in the last 12-months of life [3, 5]. While these symptoms may contain important prognostic information, they were not routinely captured in our study and therefore not included in the analysis. We have however, included the presence of dysphagia, as well as albumin and urea levels as proxy indicators for eating problems and malnutrition. Challenges with swallowing impacts a patient’s food intake and places them at a higher risk of malnutrition. Abnormal levels of albumin and urea are common markers of systemic inflammation and often manifest when patients have challenges with swallowing and malnourishment.

### Application of prognostic model

The underlying goal for this predictive algorithm is to risk-stratify patients. By assigning patients with a certain probability threshold, high-risk patients can be identified early to receive integrated palliative care while low-risk patients can continue disease-modifying treatments [39].

The application of a probability threshold of 0.7 would result in a model with low sensitivity and high specificity. Of the high-risk patients, 77.79% (true positives) referred to palliative care will die within one-year, while 22.21% (false positives) will not. On the other hand, about 73.52% (true negatives) would have been correctly identified as low-risk by the model as they will survive beyond one-year. Unfortunately, 26.48% (false negatives) of those identified to be at low risk of dying within a year would have died without a referral to palliative care.

If a lower probability threshold such as 0.3 were considered, the model would display better sensitivity. One in two patients referred to palliative care would die within one-year (true positives). Of those who were classified as low risk by the tool, about 83.31% (true negatives) would have survived more than one-year and 16.69% (false negatives) of these patients would have died within one-year.

The appropriate probability threshold depends on the acceptable ratio of error (false negatives and false positives), their implications on treatment, care plans and availability of healthcare resources. By lowering the probability score that we use to classify someone as high risk of dying, we will refer a higher proportion of the study sample for palliative care support, but some may not pass on within the next 12-months. We can do this if there are sufficient resources within the healthcare system to support the needs of these patients. Palliative care discussions can occur alongside disease-modifying treatments. Serious illness discussions can facilitate the development of a comprehensive treatment plan that is medically sound and concordant with patient’s wishes and values. When it reaches a point when disease-modifying treatments no longer improve quality of life, treatment can transition to focus on a comfort directed approach. Together with medical care teams and palliative care physicians, understanding of funding mechanisms and resource availability, these thresholds can be further deliberated to suit the needs of patients and resource availability accordingly.

### Strengths and Limitations

The current model addresses the research gap in the dearth of reliable instruments to provide accurate estimates of survival in the acute care setting where dementia care also occurs. Furthermore, to enhance clinical applicability, the model uses data collected as part of routine clinical care locally.

This study is not without limitations. The identification of AD was done retrospectively based on information documented in clinical notes. As such, we may be missing out on AD patients who had insufficient information in the clinical notes to be included in this analysis. Empirical observations in nursing homes and acute hospitals have shown that distressing symptoms such as pain and breathlessness are commonly experienced in patients with AD as death approaches [5, 40]. These variables may improve the prognostic capability of our model. However, these symptoms are not routinely documented, and therefore, were not considered during the model development stage. The variables collected were at a single time point when AD was diagnosed. We could not account for longitudinal variability in terms of function and symptoms that could contribute to prognostic information. Future studies should investigate model performance with different statistical or machine learning algorithms to account for time-varying covariates.

While the model has demonstrated good performance in this retrospective cohort, prospective validation would be helpful to further validate the usefulness of the model in the clinical setting. We noted in the external validation that a higher proportion of patients were diagnosed in the outpatient setting and this may have contributed to a higher proportion of missing data for variables such as serum urea and albumin. As some blood investigations may not be commonly done in the outpatient setting, building a separate model based on available data in this setting should be considered.

### Policy implications

In Singapore, 69% of patients with AD died in the hospital [4], suggesting that this is the predominant place of care. Although the unmet needs from physical suffering, psychosocial and emotional burden are as prevalent as patients with malignant diseases, the adoption of palliative care among AD patients is less common and initiated much later in life. Due to the challenges of recognizing the terminal phase of the illness, referrals to palliative care occur too late, resulting in missed opportunities to mitigate unnecessary suffering, to reframe care and to honour patient’s preferences for place of care based on their goals and values.

Medical care in the acute care hospital may not always be appropriate for patients in the terminal phase of illness when the primary goal of care should be directed towards palliation and improving quality of life [41]. Used synergistically with clinician judgement, this model can be used as a decision support tool to facilitate timely referrals to palliative care. This will allow well-timed discussions about the illness between patient and caregiver, ensure treatment decisions are consistent with patient’s preferences and articulated goals of care, and reduce unnecessary hospital admissions and burdensome life-prolonging interventions that do not improve quality-of-life. To mobilise and channel appropriate healthcare resources across care settings, the integration of palliative and disease-specific models of care that extends out to the community is required.

## Conclusion

The PRO-MADE is the first model in the literature that examined prognostication of AD patient presenting at an acute care setting, attaining good discrimination and calibration properties. Used together with clinician judgement, PRO-MADE can be used as a decision support tool to identify AD patients at high-risk of all-cause mortality within one-year of AD diagnosis to support timely referrals to palliative care.

## Electronic supplementary material

Below is the link to the electronic supplementary material.

Additional File 1: Two stage criteria used to identify advanced dementia patients retrospectively. Additional File 2: Potential prognostic variables. Additional File 3: Missing data and imputation results. Additional File 4: Calibration plots of imputed datasets selected at random. Additional File 5: Comparison of patients included in the development and external validation datasets. Additional File 6: Recalibrated equation

## Data Availability

Access restrictions apply to the availability of these datasets due to NHG’s data protection policies and restrictions imposed by the ethics committee to ensure data privacy of patients. As such, these datasets cannot be made publicly available. Reasonable request of aggregated data can be made to corresponding author.

## List of Abbreviations

PRO-MADE: PROgnostic Model for Advanced DEmentia
AD: Advanced dementia
LTC: Long-term-care
TTSH: Tan Tock Seng Hospital
RHS: Regional Health System
NHG: National Healthcare Group
SHS: SingHealth System
NUHS: National University Health System
ICD-10-CM: International Classification of Diseases, tenth revision, clinical modification
FAST: Functional Assessment Staging Tool
MMSE: Mini-mental state examination
IP: Inpatient
ED: Emergency Department
SOC: Specialist Outpatient Clinic
CCI: Charlson Comorbidity Index
TRIPOD: Transparent Reporting of a Multivariable Prediction Model for Individual Prognosis or Diagnosis
SD: Standard Deviation
AUC: Area Under the Curve
CITL: Calibration-in-the-large
PPV: Positive predictive value
NPV: Negative predictive value CI:Confidence Interval
Pr: Probability
