# Supplementary material for: Predicting mortality in patients diagnosed with advanced dementia presenting at an acute care hospital: the PROgnostic Model for Advanced DEmentia (PRO-MADE)
Source: BMC Geriatr. 2023 Apr 28;23:255. doi: 10.1186/s12877-023-03945-8 (PMC10148534; doi:10.1186/s12877-023-03945-8)
Supplement: Supplementary file 1 — Additional File 1: Two stage criteria used to identify advanced dementia patients retrospectively. Additional File 2: Potential prognostic variables. Additional File 3: Missing data and imputation results. Additional File 4: Calibration plots of imputed datasets selected at random. Additional File 5: Comparison of patients included in the development and external validation datasets. Additional File 6: Recalibrated equation [file 12877_2023_3945_MOESM1_ESM.docx]

**Additional File 1: Two stage criteria used to identify advanced dementia patients retrospectively**

As FAST is not readily available or used in our local setting, we employed a two-step identification strategy to identify AD patients.

1. Based on primary or secondary diagnosis of dementia, using ICD-10-CM codes, we screened for patients who were admitted to Tan Tock Seng Hospital between 1^st^ July 2016 and 31^st^ October 2017 **(Table S1)**. A total of 2,729 patients were identified to have dementia diagnosis at this stage.
2. Two trained research assistants screened through the medical notes (nursing notes, laboratory results, speech therapist notes, physiotherapy notes and discharge summary) from 1^st^ January 2005 to 31^st^ October 2017 to retrospectively identify AD patients who had characteristics consistent with Functional Assessment Staging Tool (FAST) stage seven **(Table S2)**, severe cognitive impairment defined as mini-mental state examination (MMSE) score ≤10 or had a clinical diagnosis of AD in the medical notes. 1,967 patients were screened for AD diagnosis. 890 patients were excluded as patients did not meet FAST stage seven criteria (n=779) or were already referred to palliative care, inpatient or home hospice due to poor prognosis (n=111).

**Additional File 1 Table S1: List of ICD-10CM codes for dementia**

| **ICD-10** | **List of dementia codes from literature** |
| --- | --- |
| **F00** | **Dementia in Alzheimer disease** |
| F00.0 | Dementia in Alzheimer disease with early onset |
| F00.1 | Dementia in Alzheimer disease with late onset |
| F00.2 | Dementia in Alzheimer disease, atypical or mixed type |
| F00.9 | Dementia in Alzheimer disease, unspecified |
| **F01** | **Vascular dementia** |
| F01.0 | Vascular dementia of acute onset |
| F01.1 | Multi-infarct dementia |
| F01.2 | Subcortical vascular dementia |
| F01.3 | Mixed cortical and subcortical vascular dementia |
| F01.8 | Other vascular dementia |
| F01.9 | Vascular dementia, unspecified |
| **F02** | **Dementia in other diseases classified elsewhere** |
| F02.0 | Dementia in Pick disease |
| F02.1 | Dementia in Creutzfeldt-Jakob disease |
| F02.2 | Dementia in Huntington disease |
| F02.3 | Dementia in Parkinson disease |
| F02.4 | Dementia in human immunodeficiency virus [HIV] disease |
| F02.8 | Dementia in other specified diseases classified elsewhere |
| **F03** | **Unspecified dementia** |
| **F05.1** | **Delirium superimposed on dementia** |
| **F05.0** | Delirium not superimposed on dementia, so described |
| **G30** | **Alzheimer disease** |
| G30.0 | Alzheimer disease with early onset |
| G30.1 | Alzheimer disease with late onset |

**Additional File 1 Table S2: Characteristics of FAST stage 7**

| **FAST STAGE** | **Reisberg’s (1986) Description [1]** | **Mapping of keywords found from clinical notes that were used by Research assistants (not exhaustive)** |
| --- | --- | --- |
| 7A | Speech limited to about 6 words in the course of an average day-during the course of an average day the patient’s speech is restricted to single words (e.g., “Yes,” “No,” “Please”) or short phrases (e.g., “please don’t hurt me”; “get away”; “get out of here”; “I like you”) | Having less speech; Still able to speak > 6 words; incomprehensible speech at times due to mumbling but 100% intelligible during shouting (e.g. "don't touch me", "I don't want to eat); speaks in full sentences |
| 7B | Intelligible vocabulary limited to generally a single word in the course of an average day-as the illness progresses the ability to utter even short phrases on a regular basis is lost so that the spoken vocabulary becomes limited to generally 1 or 2 single words as an indicator for all things and needs (e.g., “Yes,” “No,” “O.K.”for all verbalization-provoking phenomena); | Minimally communicative;  difficulty understanding yes/no questions - tend to reply 'ya' to all questions; Chairbound/bedbound and non-communicative for >1 year;  max 1 word (dog) (7B Since 2014); Uncommunicative; Able to give me one word answer, speaks 1-2 words; only gives 1-2 word answers |
| 7C | Ambulatory ability lost-patients gradually lose the ability to ambulate independently; in the early part of this substage they may require actual support (e.g., being physically supported by a caregiver) and physical assistance to walk, but as the substage progresses, the ability to ambulate even with assistance is lost; the onset is somewhat varied with some patients simply taking progressively smaller and slower steps-other patients begin to tilt forwards, backwards or laterally when ambulating; twisted gaits have also been noted as antecedents of ambulatory loss | ADL assisted; bed bound/ chair bound; Home-ambulant with walking stick; ADL-Dependent chairbound; ADL : 1 man assisted; ADL : 2 man assisted; Chair to bedbound |
| 7D | Ability to sit up lost-the patients lose the ability to sit up without assistance (e.g., they need some form of physical brace-an arm rest, a belt, or other brace or other special devices to keep them from sliding down in the chair) | Seated ADL engagement in supported sitting; static sitting balance (static) poor; retropulsing ++; impaired sitting and standing balance; unable to sit unsupported, increased retropulse with nil trunk control in all directions; |
| 7E | Ability to smile lost-patients are no longer observed to smile, although they do manifest other facial movements and sometimes grimace | NIL |
| 7F | Ability to hold head *up* lost-patients can no longer hold up their head unless the head is supported | Plan: Regular turning. Head up 30 degree |

1. Reisberg B: **Dementia: a systematic approach to identifying reversible causes**. *Geriatrics* 1986, **41**(4):30-46.

**Additional File 2: Potential prognostic variables**

The date when symptoms of AD was first identified or recorded was considered as date of diagnosis at either inpatient or outpatient setting and was defined as the baseline.

| **No.** | **Domain** | **Candidate predictor** | **Specification** | **Point of extraction** |
| --- | --- | --- | --- | --- |
| 1 | Individual factors | Age   - ≤85 - >85 years | Category | AD diagnosis |
| 2 | Individual factors | Gender   - Male - Female | Category | AD diagnosis |
| 3 | Individual factors | Marital status   - Single - Married - Widowed/divorced | Category | AD diagnosis |
| 4 | Individual factors | Ethnicity   - Chinese - Malay - Indian - Others | Category | AD diagnosis |
| 5 | Individual factors | Housing type   - Nursing home - Rental/1-2 room public flat - 3-5 room public flat - Private property | Category | AD diagnosis |
| 6 | Individual factors | Living situation   - Living alone - With family/friends - Nursing home | Category | AD diagnosis |
| 7 | Individual factors | Presence of caregiver   - Familial - Non-familial - Both | Category | AD diagnosis |
| 8 | Individual factors | Documentation of advance care plans   - Yes - No | Category | AD diagnosis |
| 9 | Health status | Type of dementia   - Alzheimer’s disease - Vascular disease - Mixed dementia disease - Dementia disease - Others | Category | AD diagnosis |
| 10 | Health status | FAST stage   - 7a/b - 7c - 7 d/e/f - Undetermined | Category | AD diagnosis |
| 11 | Health status | CCI   - <8 - ≥8 | Category | AD diagnosis |
| 12 | Health status | Pneumonia   - Yes - No | Category | AD diagnosis |
| 13 | Health status | Pressure ulcers   - Yes - No | Category | AD diagnosis |
| 14 | Health status | Haemoglobin (mmol/L)   - Abnormal (<13.6 ; >16.6) - Normal (13.6 to 16.6) | Category | Within 3 days of inpatient admission when AD diagnosis was made or at outpatient setting when AD diagnosis was made |
| 15 | Health status | White blood cells (X10^9^/L)   - Abnormal (<4; >10) - Normal (4 to 10) | Category | Within 3 days of inpatient admission when AD diagnosis was made or at outpatient setting when AD diagnosis was made |
| 16 | Health status | Sodium (mmol/L)   - Abnormal (<135; >145) - Normal (135 to 145) | Category | Within 3 days of inpatient admission when AD diagnosis was made or at outpatient setting when AD diagnosis was made |
| 17 | Health status | Potassium (mmol/L)   - Abnormal (<3.0; >4.5) - Normal (3.0 to 4.5) | Category | Within 3 days of inpatient admission when AD diagnosis was made or at outpatient setting when AD diagnosis was made |
| 18 | Health status | Urea (mmol/L)   - Abnormal (<2.5; >7.5) - Normal (2.5 to 7.5) | Category | Within 3 days of inpatient admission when AD diagnosis was made or at outpatient setting when AD diagnosis was made |
| 19 | Health status | Albumin (g/dL)   - Abnormal (<23g/dL) - Normal (≥23g/dL) | Category | Within 3 days of inpatient admission when AD diagnosis was made or at outpatient setting when AD diagnosis was made |
| 20 | Function | Mobility impairment requiring use of aid   - Yes - No - Bedbound | Category | AD diagnosis |
| 21 | Function | Presence of dysphagia   - Yes - No | Category | AD diagnosis |
| 22 | Function | Number of dependent ADLs (Feeding, dressing, bathing, toileting, transferring, ambulating)   - <4 dependent ADLs - ≥4 dependent ADLS | Category | AD diagnosis |
| 23 | Cognitive and mental health | History of mental health conditions (depression, anxiety, mood disorder and other mental health conditions)   - Yes - No | Category | AD diagnosis |
| 24 | Cognitive and mental health | History of delirium   - Yes - No | Category | AD diagnosis |
| 25 | Cognitive and mental health | History of BPSD   - Yes - No | Category | AD diagnosis |
| 26 | Cognitive and mental health | History of agitation   - Yes - No | Category | AD diagnosis |
| 27 | Treatment | Use of enteral tube   - Yes - No | Category | AD diagnosis |
| 28 | Treatment | Prescribed with psychotropic   - Yes - No | Category | AD diagnosis |
| 29 | Treatment | Prescribed with opioids   - Yes - No | Category | AD diagnosis |
| 30 | Treatment | Prescribed with acetylcholinesterase inhibitors (ACEi)   - Yes - No | Category | AD diagnosis |
| 31 | Treatment | Prescribed with memantine   - Yes - No | Category | AD diagnosis |
| 32 | Health system factors | Referral to community or home based programmes   - Yes - No | Category | AD diagnosis |
| 33 | Health system factors | Inpatient admission   - Yes - No | Category | One year prior to AD diagnosis |
| 34 | Health system factors | Emergency department   - Yes - No | Category | One year prior to AD diagnosis |
| 35 | Health system factors | Specialist outpatient clinic   - Yes - No | Category | One year prior to AD diagnosis |
| 36 | Health system factors | Polyclinic   - Yes - No | Category | One year prior to AD diagnosis |
| 37 | Health system factors | Average length of stay (ALOS) (days) | Continuous | One year prior to AD diagnosis |

**Additional File 3: Missing data and imputation results**

We assessed the proportion of missing data for all 1,077 patients, by outcome (survivors and decedents) and by care setting where AD was diagnosed (inpatient and outpatient).

We saw that, decedents had lower % of missing for living with others, ADLs, CCI, living with others, presence of caregiver, and biochemical data. The difference was larger when stratified by care settings. There were more missing data in patients who were diagnosed in outpatient setting. Being diagnosed in the inpatient setting had lesser odds of having missing variables.

**Additional File 3 Table S1: Proportion of missing data**

| Variables with missing values | All (n=1,077)  (%) | Outcome | | Care setting | |
| --- | --- | --- | --- | --- | --- |
|  |  | Survivors (n=759)  (%) | Decedents (n=318)  (%) | Outpatient (n=136)  (%) | Inpatient (n=941)  (%) |
| Use of enteral tube | 1 | 0.9 | 1.3 | 2.9 | 0.7 |
| Pressure ulcers | 1.6 | 1.7 | 1.3 | 5.9 | 1 |
| Living situation | 2 | 2.4 | 1.3 | 2.9 | 1.9 |
| Number of dependent ADLs | 2.9 | 3.6 | 1.3 | 19.8 | 0.4 |
| CCI | 3.3 | 3.8 | 2.2 | 8.1 | 2.7 |
| Marital status | 4.7 | 4.9 | 4.4 | 6.6 | 4.5 |
| Presence of caregiver | 7.9 | 9.3 | 4.4 | 8.1 | 7.9 |
| Sodium (mmol/L) | 10.7 | 11.5 | 8.8 | 66.9 | 2.5 |
| Potassium (mmol/L) | 11 | 11.9 | 8.8 | 69.8 | 2.4 |
| Haemoglobin (mmol/L) | 11.1 | 12.2 | 8.2 | 73.5 | 2 |
| White blood cells (X10^9^/L) | 11.1 | 12.2 | 8.2 | 73.5 | 2 |
| Urea (mmol/L) | 12.3 | 13.7 | 9.1 | 76.5 | 3.1 |
| Albumin (g/dL) | 20.6 | 22.8 | 15.4 | 88.2 | 10.8 |

We imputed the missing variables and assessed for convergence across the 50 datasets.

**Additional File 3 Table S2: Convergence plots**

| **Variables** | **Convergence plots (mean)** | **Variables** | **Convergence plots (mean)** |
| --- | --- | --- | --- |
| Sodium (mmol/L) |  | White blood cells (X10^9^/L) |  |
| Potassium (mmol/L) |  | Urea (mmol/L) |  |
| Haemoglobin (mmol/L) |  | Albumin (g/dL) |  |

**Additional File 3 Table S3: Distribution of categories across imputed datasets**

We looked at the distribution of categories across the 50 imputed datasets. The proportions were similar.

| **Variables** | **Distribution of categories** | **Variables** | **Distribution of categories** |
| --- | --- | --- | --- |
| Sodium (mmol/L) |  | White blood cells (X10^9^/L) |  |
| Potassium (mmol/L) |  | Urea (mmol/L) |  |
| Haemoglobin (mmol/L) |  | Albumin (g/dL) |  |

**Additional File 4: Calibration plots of imputed datasets selected at random**

| **m** | **Calibration plots** | **m** | **Calibration plots** | |
| --- | --- | --- | --- | --- |
| **10** | **** | **40** | **** |  |
| **20** | **** | **50** | **** |  |
| **30** | **** |  |  |  |

**Additional File 5: Comparison of patients included in the development and external validation datasets.**

| **Characteristics** | **Development (n=1,077)** | **External**  **(n=550)** | ***P* value** |
| --- | --- | --- | --- |
| 1. **Individual Factors** | | | |
| **Age>85 years (col %)** | 51.5 | 55.3 | 0.51 |
| **Female (col %)** | 64.2 | 60.5 | 0.14 |
| **Marital Status (col %)**  Single  Married  Widowed/divorced  Missing data | 3.3  72.1  19.9  4.7 | 5.5  62.2  26.2  6.2 | **0.001** |
| **Ethnicity (col %)**  Chinese  Malay  Indian  Others | 87.2  4.7  7.1  1.0 | 90.5  5.6  3.5  0.4 | **0.01** |
| **Housing type (col %)**  Nursing home  Rental/1 to 2 room public flat  3 to 5 room public flat  Private property | 21.2  2.1  62.3  14.4 | 24.0  2.9  57.8  15.3 | 0.31 |
| **Living situation (col %)**  Alone  Family or friends  Nursing home  Missing data | 4.7  72.1  21.1  2.0 | 5.3  70.0  24.0  0.7 | 0.12 |
| **Presence of caregiver (col %)**  Familial  Non-familial  Both  Missing data | 19.5  70.1  2.5  7.9 | 17.1  74.4  3.3  5.3 | 0.09 |
| **Documentation of ACP (col %)** | 35.0 | 39.8 | 0.06 |
| 1. **Health Status** | | | |
| **Dementia type (col %)**  Alzheimer’s disease  Vascular disease  Mixed dementia disease  Dementia disease  Others | 31.1  25.3  22.9  18.6  2.0 | 39.5  23.6  14.0  18.2  4.7 | **<0.001** |
| **FAST stage (col %)**  7a/b  7c  7 d/e/f  Undetermined | 3.0  92.5  1.0  3.5 | 1.3  91.8  2.2  4.7 | **0.027** |
| **CCI (col %)**  <8  ≥8  Missing data | 40.8  55.8  3.3 | 59.3  39.8  0.9 | **<0.001** |
| **Pneumonia (col %)**  Yes  No | 23.1  76.9 | 22.7  77.3 | 0.86 |
| **Pressure ulcers (col %)**  Yes  No  Missing data | 8.4  90.0  1.6 | 18.4  67.3  14.4 | **<0.001** |
| **Haemoglobin (mmol/L) (col %)**  Normal (13.6 to 16.6)  Abnormal (<13.6 ; >16.6)  Missing data | 11.5  77.4  11.1 | 13.4  60.0  26.6 | **<0.001** |
| **White blood cells (X10^9^/L) (col %)**  Normal (4 to 10)  Abnormal (<4; >10)  Missing data | 43.4  40.6  11.1 | 43.3  30.2  26.5 | **<0.001** |
| **Sodium (mmol/L) (col %)**  Normal (135 to 145)  Abnormal (<135; >145)  Missing data | 56.8  32.5  10.7 | 48.7  25.1  26.2 | **<0.001** |
| **Potassium (mmol/L) (col %)**  Normal (3.0 to 4.5)  Abnormal (<3.0; >4.5)  Missing data | 71.1  179  11.0 | 58.9  14.2  26.9 | **<0.001** |
| **Urea (mmol/L) (col %)**  Normal (2.5 to 7.5)  Abnormal (<2.5; >7.5)  Missing data | 47.0  40.7  12.3 | 36.9  34.9  28.2 | **<0.001** |
| **Albumin (g/dL) (col %)**  Normal (≥23g/dL)  Abnormal (<23g/dL)  Missing data | 72.8  6.6  20.6 | 63.1  2.0  34.9 | **<0.001** |
| 1. **Function** | | | |
| **Mobility impairment requiring use of aid (col %)**  Yes  No  Bedbound | 56.5  7.2  36.2 | 68.4  10.2  21.5 | **<0.001** |
| **Presence of dysphagia (col %)** | 48.7 | 42.4 | **0.016** |
| **Number of dependent ADLs (col %)**  < 4  ≥ 4  Missing data | 67.9  29.2  2.9 | 72.4  24.4  3.3 | 0.111 |
| 1. **Cognitive and mental health** | | | |
| **History of depression, anxiety, mood disorders and other conditions related to mental health (col %)** | 10.7 | 6.7 | **0.01** |
| **History of delirium (col %)** | 27.9 | 17.1 | **<0.001** |
| **History of BPSD (col %)** | 25.6 | 22.2 | 0.13 |
| **History of agitation (col %)** | 11.7 | 13.8 | 0.22 |
| 1. **Treatment** | | | |
| **Use of enteral tube (col %)**  Yes  No  Missing data | 15.2  83.7  1.0 | 84.5  14.4  1.1 | 0.89 |
| **Prescribed with psychotropic (col %)** | 45.2 | 42.9 | 0.37 |
| **Prescribed with opioids (col %)** | 5.7 | 4.2 | 0.20 |
| **Prescribed with ACEi (col %)** | 4.8 | 10.9 | **<0.001** |
| **Prescribed with memantine (col %)** | 6.0 | 6.9 | 0.49 |
| 1. **Health system factors** | | | |
| **Referrals to community or home-based programs (col %)** | 20.3 | 16.7 | 0.08 |
| **Had IP (col %)** | 88.3 | 90.5 | 0.17 |
| **Had ED (col %)** | 95.2 | 91.4 | **0.003** |
| **Had SOC (col %)** | 71.0 | 76.9 | **0.012** |
| **Had Polyclinic (col %)** | 41.8 | 36.4 | **0.035** |
| **ALOS (days) (mean, SD)** | 30.1 (37.6) | 27.4 (33.2) | 0.16 |

**Additional File 6: Recalibrated equation**

We performed recalibration, where the model was refitted, and coefficients were updated. Overall model performance (Nagelkerke’s R^2^: 0.18 (95% CI: 0.17 to 0.18; Brier’s score: 0.17 (95% CI: 0.17 to 0.17), discrimination (AUC: 0.73 (95% CI: 0.72 to 0.73) and calibration (slope: 1; CITL: 0) properties of the model improved.

Recalibrated equation is as follows:

logit(p) = -2.33 + 0.55*Age>85years + 0.17*Male + 0.89*Pneumonia + 0.76*Pressure Ulcers + 0.62*CCI≥8 + 0.36*ADL≥4 functional dependencies + 0.02*Dysphagia+ 0.16*Abnormal Urea + 1.37*Abnormal Albumin

**Sensitivity, Specificity, PPV and NPV at different probability thresholds***

| **Pr** | **Sensitivity (%)** | **Specificity (%)** | **PPV (%)** | **NPV (%)** |
| --- | --- | --- | --- | --- |
| **0.9** | 0.55  (95% CI: 0.34 to 0.76) | 99.97  (95% CI: 99.92 to 100.0) | 93.75  (95% CI: 78.97 to 100.0) | 73.74  (95% CI: 73.70 to 73.78) |
| **0.8** | 1.03  (95% CI: 0.77 to 1.29) | 99.83  (95% CI: 99.74 to 99.91) | 73.33  (95% CI: 59.33 to 87.33) | 73.80  (95% CI: 73.76 to 73.84) |
| **0.7** | 3.93  (95% CI: 3.12 to 4.74) | 99.16  (95% CI: 99.01 to 99.31) | 62.5  (95% CI: 61.09 to 63.90) | 74.25  (95% CI: 74.11 to 74.38) |
| **0.6** | 11.38  (95% CI: 9.19 to 13.56) | 97.97  (95% CI: 97.54 to 98.41) | 67.05  (95% CI: 64.75 to 69.362) | 75.54  (95% CI: 75.16 to 75.93) |
| **0.5** | 29.24  (95% CI: 28.05 to 30.43) | 92.22  (95% CI: 91.96 to 92.48) | 57.36  (95% CI: 56.64 to 58.09) | 78.45  (95% CI: 78.20 to 78.70) |
| **0.4** | 39.45  (95% CI: 38.34 to 40.56) | 88.54  (95% CI: 88.23 to 88.86) | 55.21  (95% CI: 54.27 to 56.15) | 80.33  (95% CI: 80.04 to 80.62) |
| **0.3** | 52.83  (95% CI: 51.95 to 53.70) | 78.94  (95% CI: 78.21 to 79.66) | 47.33  (95% CI: 46.36 to 48.31) | 82.37  (95% CI: 82.07 to 82.68) |
| **0.2** | 76.90  (95% CI: 75.99 to 77.80) | 55.93  (95% CI: 54.96 to 56.89) | 38.46  (95% CI: 37.91 to 39.00) | 87.11  (95% CI: 86.66 to 87.57) |
| **0.1** | 97.31  (95% CI: 96.77 to 97.85) | 10.47  (95% CI: 8.77 to 12.17) | 28.02  (95% CI: 27.71 to 28.33) | 91.57  (90.49 to 92.66) |

** Proportion of AD patients who died within one-year: 26.4%*

*Pr: Probability; 95% CI: 95% Confidence Interval*
